# Novel measurement of spreading pattern of influenza epidemic by using weighted standard distance method: retrospective spatial statistical study of influenza, Japan, 1999–2009

**DOI:** 10.1186/1476-072X-11-20

**Published:** 2012-06-19

**Authors:** Yugo Shobugawa, Seth A Wiafe, Reiko Saito, Tsubasa Suzuki, Shinako Inaida, Kiyosu Taniguchi, Hiroshi Suzuki

**Affiliations:** 1 School of Public Health, Loma Linda University, 24760 Stewart St. CC 3104, Loma Linda, CA 92350, USA; 2 Department of International Health, Niigata University Graduate School of Medical and Dental Sciences, 1-757 Asahimachi-dori, Chuo district, Niigata city 951-8510, Japan; 3 Infectious Disease Surveillance Center, National Institute of Infectious Diseases, 1-23-1 Toyama, Shinjuku district, Tokyo, 162-8640, Japan; 4 Faculty of Nursing, Social Welfare, and Psychology, Niigata Seiryo University, 1-5939 Suido cho, Chuo district, Niigata city 951-8121, Japan

**Keywords:** Weighted standard distance, Influenza, Spatial compactness

## Abstract

**Background:**

Annual influenza epidemics occur worldwide resulting in considerable morbidity and mortality. Spreading pattern of influenza is not well understood because it is often hampered by the quality of surveillance data that limits the reliability of analysis. In Japan, influenza is reported on a weekly basis from 5,000 hospitals and clinics nationwide under the scheme of the National Infectious Disease Surveillance. The collected data are available to the public as weekly reports which were summarized into number of patient visits per hospital or clinic in each of the 47 prefectures. From this surveillance data, we analyzed the spatial spreading patterns of influenza epidemics using weekly weighted standard distance (WSD) from the 1999/2000 through 2008/2009 influenza seasons in Japan. WSD is a single numerical value representing the spatial compactness of influenza outbreak, which is small in case of clustered distribution and large in case of dispersed distribution.

**Results:**

We demonstrated that the weekly WSD value or the measure of spatial compactness of the distribution of reported influenza cases, decreased to its lowest value before each epidemic peak in nine out of ten seasons analyzed. The duration between the lowest WSD week and the peak week of influenza cases ranged from minus one week to twenty weeks. The duration showed significant negative association with the proportion of influenza A/H3N2 cases in early phase of each outbreak (correlation coefficient was −0.75, *P* = 0.012) and significant positive association with the proportion of influenza B cases in the early phase (correlation coefficient was 0.64, *P* = 0.045), but positively correlated with the proportion of influenza A/H1N1 strain cases (statistically not significant). It is assumed that the lowest WSD values just before influenza peaks are due to local outbreak which results in small standard distance values. As influenza cases disperse nationwide and an epidemic reaches its peak, WSD value changed to be a progressively increasing.

**Conclusions:**

The spatial distribution of nationwide influenza outbreak was measured by using a novel WSD method. We showed that spreading rate varied by type and subtypes of influenza virus using WSD as a spatial indicator. This study is the first to show a relationship between influenza epidemic trend by type/subtype and spatial distribution of influenza nationwide in Japan.

## Background

Despite advances in medical treatment, annual influenza epidemics affect 10–20% of the population resulting in about three to five million cases of severe illness and about 250,000 to 500,000 deaths worldwide [1]. Consequently, influenza has a considerable public health impact. The epidemic of influenza in temperate zones is characterized by seasonal cycles with marked peaks in winter [2]. The spreading pattern of influenza is not well elucidated because it is often hampered by the quality of surveillance data that limits reliability of analysis. In Japan, influenza is a notifiable disease, which is reported on a weekly basis from 5,000 hospitals and clinics nationwide under the scheme of Infectious Disease Surveillance Center (IDSC). The collected data are open to public as weekly reports which were aggregated into number of patient visits per hospital or clinic in each of the 47 prefectures.

A few other studies tried to analyze geographical spreading pattern of influenza [3,4]. In our previous study, we showed nationwide geographic influenza peak trend using kriging method [5]. However, spreading pattern or distribution of influenza was not well elucidated. In this study, we analyzed the spread and distribution pattern of influenza by using a spatial indicator; Weighted Standard Distance (WSD), which measures the spatial compactness of distribution. Our analysis of the weekly surveillance data generated from April 1999 to June 2009 showed that the WSD method could simplify the influenza distribution pattern as a numerical value to evaluate the spatial trend of the epidemic. WSD significantly decreased to a minimum value before influenza epidemic peak and then increased during the epidemic and inter-epidemic period, and this pattern varied according to type and subtype of influenza virus.

## Results

### Influenza epidemics, 1999–2009

Weekly influenza incidence values were analyzed using national influenza surveillance data from ten influenza seasons between 1999 through 2009. Annual influenza seasons started between November and December and peaked between January and February before returning to baseline between April and June for the study period. The predominant circulating influenza strains in early phase of epidemic varied by season (Table 1). A strain is considered predominant if it is more than 50% of the total detected strains in the early phase. Early phase was defined as the period from the detection of first laboratory-confirmed until the peak week of influenza surveillance cases. Influenza A/H3N2 was the dominant subtype during 2002/2003, 2003/2004, and 2005/2006 seasons, while influenza A/H1N1 dominated the 1999/2000, 2001/2002, 2007/2008, and 2008/2009 seasons. Only in the 2004/2005 season was the influenza B virus the dominant strain in early phase of epidemic. Circulating A/H3N2 strain’s antigenicity changed frequently and resulted in five updates of the vaccine strain: strain A/Sydney/5/97, A/Panama/2007/99 in 2000/2001, A/Fujian/411/2002 in 2002/2003, A/California/7/2004 in 2004/2005, A/Wisconsin/67/2005 in 2005/2006, and A/Brisbane/10/2007 in 2006/2007 [6]. On the contrary, the main circulating A/H1N1 strain varied only once in 10 seasons from A/New Caledonia/20/99 to A/Solomon Islands/3/2006 in the 2006/2007 season [6].

**Table 1:** Relationship between duration week from the lowest WSD week to the peak week and proportions of circulated type/subtype of influenza virus

**Seasons**	**Lowest WSD week (a)**	Peak week* (b)	**Duration between (a) and (b), weeks**	**Prevalence of type or subtype of influenza virus in early phase of the season†**					
					**A/H3N2**		**A/H1N1**		**B**
1999/2000	51st, 1999	5th, 2000	6	45.2%	A/Sydney/5/97	54.8%	A/New Caledonia/20/99	0.0%	-
2000/2001	48th, 2000	10th, 2001	14	14.3%	A/Panama/2007/99	43.4%	A/New Caledonia/20/99	42.3%	B/Sichuan/379/99
2001/2002	1st, 2002	8th, 2002	7	38.3%	A/Panama/2007/99	51.2%	A/New Caledonia/20/99	10.5%	B/Shandong/7/97
2002/2003	52nd, 2002	5th, 2003	5	85.4%	A/Fujian/411/2002	0.0%	-‡	14.6%	B/Shandong/7/97
2003/2004	52nd, 2003	6th,2004	6	98.6%	A/Fujian/411/2002	0.1%	-	1.3%	B/Shanghai/361/2002
2004/2005	42nd, 2004	9th, 2005	20	35.4%	A/California/7/2004	3.7%	A/New Caledonia/20/99	60.9%	B/Shanghai/361/2002
2005/2006	6th, 2006	5th,2006	-1§	83.0%	A/Wisconsin/67/2005	16.1%	A/New Caledonia/20/99	0.9%	B/Malaysia/2506/2004
2006/2007	49th, 2006	11th, 2007	14	49.0%	A/Brisbane/10/2007	9.2%	A/New Caledonia/20/99 ∣ A/Solomon Islands/3/2006	41.8%	B/Malaysia/2506/2004
2007/2008	39th, 2007	5th, 2008	18	5.4%	A/Brisbane/10/2007	93.0%	A/Solomon Islands/3/2006	1.6%	B/Florida/4/2006
2008/2009	44th, 2008	5th, 2009	13	28.1%	A/Brisbane/10/2007	61.8%	A/Solomon Islands/3/2006	10.1%	Brisbane/60/2008

* The peak week of influenza cases.

† See method section about a definition of “early phase”.

‡ Data not available.

§ Minus value of duration between means that peak week came earlier than the lowest WSD week.

∣ After A/New Caledonia/20/99 was circulated, A/Solomon Islands/3/2006 was equivalently circulated in 2006/2007 season.

### Spatial analyses

**Figure 1:**
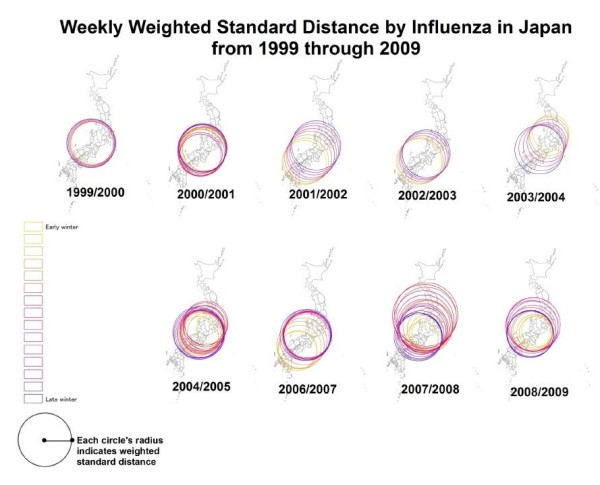
**Weekly weighted standard distance by influenza cases in Japan, from 1999 through 2009.** Each map represents the weekly WSD circles calculated for the 1999/2000 through 2008/2009 winter seasons except 2005/2006 season and range from the lowest WSD week (early) to the peak epidemic week (late). The reason for missing map in 2005/2006 was that the peak week came before the lowest WSD week. Early week values are represented by yellow color circles while subsequent weeks are represented by a progressive color scale (changing from orange to red, pink, purple, and blue). Central points of each circle represent geographic mean centers for all prefectures weighted by the magnitude of influenza activity.

**Figure 2:**
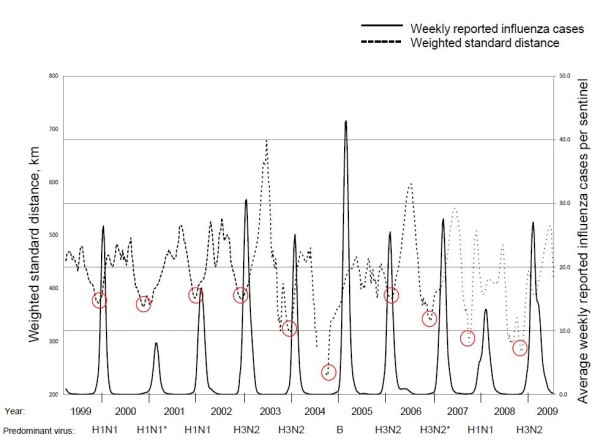
**Relationship between influenza epidemic trend and WSD trend.** The fine dashed line represents WSD values (kilometers) while the solid, bold line represents the number of weekly influenza cases per sentinel. Red circles were drawn around the lowest WSD weeks of each epidemic season. Predominant influenza type and subtype were shown below the year. Predominant strains were defined by accounting to more than 50% of total detected strains in the early phase. *In 2000/2001 and in 2006/2007, no strain accounted to more than 50% of total detected strains, thus, the majority strain was shown, alternatively.

Weighted distributions over time were evaluated to elucidate the dynamic manner of nationwide influenza outbreaks throughout Japan. We employed the WSD method to evaluate the spatial compactness of the weekly influenza case distribution (See method section about how to calculate WSD value). Weekly WSD values were represented with colored circles and overlaid on national maps using ArcGIS 10 (ESRI, Redlands, CA) for each season between the lowest WSD week and the peak week (named “early” and “late” respectively) (Figure 1). Yellow circle has the smallest radius in respective season, which represents the lowest WSD week. Circle size generally increased weekly from the lowest WSD week to the peak week. Center of circle was defined by weighted mean center of each week (See method section about weighted mean center). Typically, WSD values decreased to the lowest value before the peak week in all influenza seasons (Figure 2). In the 2005/2006 season, however, the lowest WSD value was observed in the week directly after the season’s peak. After reaching minimum value of WSD, it changed to be increasing through peak epidemic and to inter-epidemic period. WSD value was close to 400 km at each of the peak week. During the inter-epidemic period, the WSD value was high and fluctuating, then, it gradually decreased before the peak epidemic. An animated clip of the progression of WSD values over time is available as the Additional file 1.

### Statistical analyses

**Figure 3:**
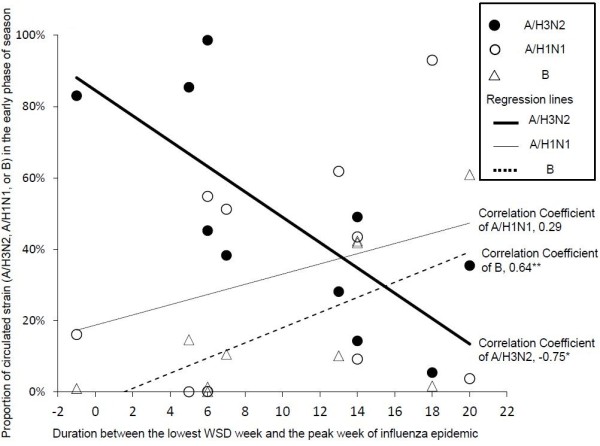
**Association between the duration weeks and the proportions of circulated influenza type/subtype.** The relationship between duration weeks (x axis) and the proportion of types and subtypes of circulated influenza virus (y axis) are represented in the technical graph. *Significant negative association with the proportion of influenza A/H3N2 cases in early phase of the epidemic (correlation coefficient was −0.75 with *P* value = 0.012), and **significant positive association with the proportion of influenza B cases in early phase of the epidemic (correlation coefficient was 0.64 with *P* value = 0.045).

The duration between two set points, namely, the lowest WSD week and the peak week of influenza epidemic ranged from minus one to plus twenty weeks (Table 1). Minus value means the peak week came before the lowest WSD week. The length of duration (in weeks) showed significant negative association with the proportion of influenza A/H3N2 cases in the early phase of the epidemic (correlation coefficient was −0.75 with *P* value = 0.012) (Figure 3), and showed significant positive association with the proportion of influenza B cases (correlation coefficient was 0.64 with *P* value = 0.045). Values were positively correlated with the proportion of influenza A/H1N1 strain cases, but correlation coefficient values were 0.29 with *P* value = 0.409.

## Discussion

It is empirically known that, prior to a national epidemic, small sporadic influenza outbreaks occur widely. Various factors such as social and environmental factors i.e., temperature and humidity [7,8], host immunity by vaccination [9,10] and previous infection, human transportation [11-13], and population density [14] may affect whether certain local outbreaks can successfully trigger a national outbreak. We demonstrated that WSD values decreased to the lowest value before each epidemic peak then increased in inter epidemic period, and this trend was observed in nine of ten influenza seasons. The weighted standard distance method is useful for measuring the compactness of geographic distribution as shown in this study. This method is the classical way to assess distribution pattern: localized or diversified, however, very few application case was found in research study [15]. Also, only a limited study was found in the literature which utilized spatial statistical method to clarify the spreading pattern in infectious diseases [16-18]. WSD values represent spatial distributions of influenza outbreak as numerical data: clustered distribution is expressed as small value; dispersed one is represented as large value. We postulated that the reason for a decrease in WSD values before the peak in influenza activity is due to local outbreak and clustering in a limited region which results in a small standard distance value. Furthermore, when successive cases disperse nationwide, the influenza outbreak peaks, thus resulting in an increasing WSD value. Also in inter epidemic season, sporadic imported cases with travelers and visitors might result in increased WSD value. This study is the first to show a spatial measurement to evaluate compactness of the distributions of influenza outbreak nationwide with WSD method. In the current study, we combined the number of reported influenza cases and spatial indicator calculated by the distribution of influenza cases, and found a significant association between these two factors. This is a novel measurement of nationwide influenza outbreak in Japan which can be applied for influenza epidemiology.

We found that the duration between the lowest WSD week and the peak week, reflected a significantly negative correlation with the proportion of A/H3N2 subtypes, and a significantly positive correlation with the proportion of type B viruses, in early phase of the each corresponding season. These results suggest that higher prevalence of A/H3N2, the predominant epidemic strain, may correlate with a faster diffusion from the lowest WSD week to the peak of a nationwide epidemic, and that B strain correlate with a slower diffusion from the lowest WSD week to the peak of a nationwide epidemic. Other studies employed spatial statistics tools to analyze spreading patterns and to trace infectious agents [17,18]. Although those studies have shown distribution pattern using spatial tool and they did not find any significant associations between spatial component and magnitude of the epidemic.

The fastest spreading epidemic was observed during the 2002/2003 influenza season with the new antigenic variants of A/H3N2. This was also shown in our previous report through a kriging method [5]. Faster evolution of influenza A/H3N2 may contribute to increased susceptibility among the population, thus accelerating the rate of epidemic spread [8,19]. In addition, influenza A/H3N2 virus causes the most severe symptoms for human among the three types and subtypes of influenza; A/H3N2, A/H1N1, and B [20]. From a clinical standpoint, patients infected with A/H3N2 virus could shed more virus and spread the infection [21]. As a result, rapid A/H3N2 spreading patterns at the community level may lead to a rapid spread at the national level. However, cause of different spreading patterns among type and subtype is still unclear.

In the 2005/2006 season, the peak epidemic week came atypically before the lowest WSD week. In this season, A/H3N2 was the predominant circulating strain (64.8%) in the early phase of epidemic. Perhaps, very large and fast outbreak occurred in the clustered area in the early phase of epidemic, thus peak epidemic and clustering occurred simultaneously. Further detailed research study is needed to elucidate timing and location for local clustering in the early phase of epidemic.

In inter-epidemic periods, unstable WSD trends were observed. These trends may be the result of fluctuating and sporadic influenza cases in local areas due to imported cases from abroad. Easily changeable WSD values, especially in inter-epidemic period, might not be reliable for analysis. In order to decrease influence by these fluctuating values, we adjusted the data with un-weighted moving average method.

The WSD represents compactness of influenza epidemics except in cases of clustered distributions at opposite sides of the study area. For instance, if a local outbreak was clustered in different areas at the same time (e.g. in far north and far south); this may result to a large WSD value. However, based on the successive ten-year national influenza surveillance data, we found that the WSD value reached a minimum just before the peak epidemic week indicating the presence of certain local clusters prior to each seasonal epidemic. The WSD method has proven valuable for the analysis of influenza outbreaks throughout Japan due to the country’s unique and isolated geography with limited entrance routes. Further studies are needed to apply the result for continental countries as well as the temperate and tropical/subtropical zones, and also needed to find different characteristic of WSD trend in different geographical condition.

In our analysis, we evaluated only compactness by using standard distance method. In Figure 1, circles moved weekly depending on the locations of weighted mean center. Those circles’ movement does not match the result of the previous study [5]. However, the method was different among this study and the previous one. The previous study focused on only the peak week of the epidemic. On the other hand, our study focused distribution of weekly influenza cases over time.

One of the limitations of our study is that we did not take social and environmental factors into consideration. Socio-economic factors might affect influenza spreading pattern [22]. However, we could not obtain enough socio-economic information to analyze this time. We plan to conduct a study to evaluate those impacts of surrounding factors nationwide. However, it may be more appropriate to perform detailed analysis in focused area including social and environmental factors. For example, dispersion pattern may be different with regional areas if early outbreak occurs in major metropolitan areas such as Tokyo and Osaka which have function of transportation hub.

A limitation of using influenza surveillance data in this study is reflected in the lack of conclusive laboratory diagnosis that divides the cases by influenza strain. For more accurate analysis, more research must be done on data that clearly diagnoses the specific viral strain. There are some more limitations in our study. As we can exactly recognize when is the lowest WSD week only after epidemic was over, we cannot predict the timing of starting using the WSD value in the middle of epidemic. Interestingly WSD value was close to 400 km when influenza epidemic peaked, which may be a kind of threshold value. However, even this method cannot predict the timing of peak epidemic because WSD value exceeded 400 km in several times before the peak epidemic. Further study is needed to predict the timing of onset and peak of influenza epidemic nationwide.

Another limitation is from elongate shape of Japan islands. Location of weighted mean center which is calculated by using each location of points (centroid of each prefecture) affects WSD value. Clustered outbreak far from mean center tend to be underestimated resulting as large WSD value, and vice versa. However, we expressed a compactness of nationwide influenza distribution as a whole, not focusing on only local region. Even though WSD values were affected by distance from mean center, WSD values were still meaningful because those trends were clearly generalized in this study and had significant relationship with influenza epidemic trend. If there is better way to evaluate skewed distribution, more exact analysis of spatial pattern of influenza epidemic could be done, further detailed study is crucially needed.

In our analysis, we did not employ data from the 2009/2010 pandemic H1N1 influenza season due to excessive influenza reporting during this time. Analysis of this pandemic season should be performed separate and could give interesting insight on the pandemic spreading patterns compared to seasonal outbreaks. We plan to perform a transmission analysis for pandemic H1N1 influenza virus (A/H1N1pdm) in the near future.

## Conclusions

The WSD method shown in this study is better to understand spatial spreading pattern of influenza depending on strains. This measure gives a new approach to evaluate spatial dynamic trend from the statistical view, to apply not only for influenza but also other infectious diseases. It might be helpful and beneficial from the public health perspective. Spatial statistics using GIS can be powerful tool to analyze and study spread of infectious diseases.

## Methods

### Data sources

All data used in this study are available to the public from the weekly report of infectious diseases; Infectious Diseases Weekly Report: IDWR (http://idsc.nih.go.jp/idwr/index-e.html) and Infectious Agents Surveillance Report: IASR (http://idsc.nih.go.jp/iasr/index.html). The data were collected under the scheme of National Infectious Disease Surveillance, which is legally defined and implemented by the Ministry of Health, Welfare, and Labor in Japan. Influenza is a notifiable disease under Japan’s Infectious Disease Control Law since 1992, which started as the Influenza-like illness report from 1981. Influenza is defined by the following symptoms: sudden onset of fever, general fatigue, headache, or myalgia. The number of patients with influenza is reported on a weekly basis from ~5,000 sentinel pediatric and general physician’s clinics or hospitals to 500 public health centers throughout Japan. The number of sentinels assigned to each public health service area is determined based on the population size: a public health center with less than 75,000 individuals would have one sentinel, between 75,000 and 125,000 individuals would have two sentinels, and greater than 125,000 individuals would have three or more [3 + (population – 125,000)/100,000] sentinels. Clinical data from each sentinel is forwarded to public health centers where the generated data is then electronically reported to ~60 prefectural or municipal public health institutes as well as the Infectious Disease Surveillance Center (IDSC) in the National Institute of Infectious Diseases (Tokyo). The IDSC also receives pathogen identification reports from prefectural and municipal public health institutes and quarantine stations, and receives infectious disease case reports from public health centers in prefectures and cities designated by ordinance. Both datasets are combined and tabulated into determined formats, analyzed, and evaluated for their public health implications. Resulting information is published as the IDWR and the IASR as shown above. IDSC opens those data to public and permitted to use the data for public health and academic research. We utilized only those publicized data on the website for this study.

### Spatial analyses

We analyzed national surveillance data from the 1999 through 2009 influenza seasons for 46 prefectures throughout Japan excluding the Okinawa Prefecture, which is an archipelago that is approximately located 800 km south of mainland Japan. By combining the data from all prefectures and understanding trends at the national level, we calculated the number of reported influenza cases per sentinel per week after adjusting for a 5-week un-weighted moving average as an indicator of influenza activity according to the former study [5]. This statistical method smoothed the resulting data and simplified the identification of seasonal influenza activity patterns. To evaluate the spatial spreading pattern of influenza infections, we employed a weighted standard distance (WSD) method to measure the spatial compactness of the influenza case distribution [23]. WSD is essentially the average distance between the weighted mean center ($\bar{X}$*,*$\bar{Y}$) and each prefectural centroid (*X*_*i*_*, Y*_*i*_*)*, and it provides a single unit measure of the spread or dispersion of a distribution. WSD was calculated with each x-coordinate (longitude) and each y-coordinate (latitude) of prefectural centroid and influenza cases in each prefecture in a certain week. As a first step, weighted mean center was calculated as below;

The weighted mean x-coordinate:

(1) $$\bar{X}=\frac{\sum_{i=1}^{n}\left(w_{i}X_{i}\right)}{\sum_{i=1}^{n}w_{i}}$$

The weighted mean y-coordinate:

(2) $$\bar{Y}=\frac{\sum_{i=1}^{n}\left(w_{i}Y_{i}\right)}{\sum_{i=1}^{n}w_{i}}$$

Multiply each *X*_*i*_ or *Y*_*i*_ (small “*i*” represents each individual prefecture) by that prefecture’s influenza cases (*w*_*i*_), then sum the weighted coordinate values, and divide the summed influenza cases. As a next step, WSD would be calculated by the weighted mean center ($\bar{X}$*,*$\bar{Y}$*)* and each prefectural centroid (*X*_*i*_*, Y*_*i*_). Equation (3) is as below:

(3) $$\text{WSD}=\sqrt{\frac{{\sum_{i=1}^{n}w_{i}\left(X_{i}-\bar{X}\right)}^{2}+\sum_{i=1}^{n}w_{i}{\left(Y_{i}-\bar{Y}\right)}^{2}}{\sum_{i=1}^{n}w_{i}}}$$

**Figure 4:**
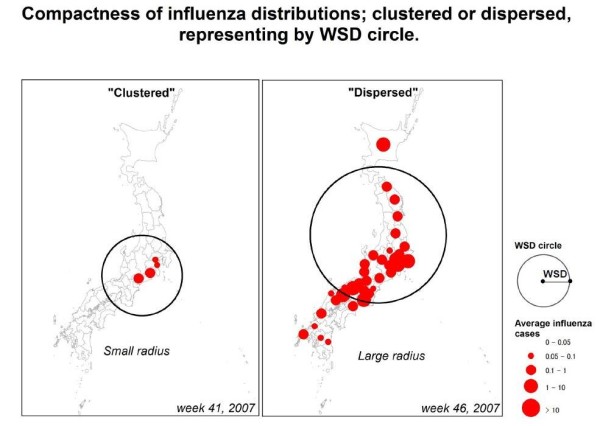
**Compactness of influenza distributions representing by WSD circle.** Comparison between typical clustered and dispersed distribution of influenza cases is shown. Left panel shows clustered pattern with small WSD value and right one shows dispersed pattern with large WSD. Radius of circle is equivalent to WSD value (unit: kilometers).

This equation uses the squared difference of coordinate values (the value between each prefectural centroid and the weighted mean center) and multiplies it by influenza cases in each prefecture, sums the weighted differences and then divides the summed values by the sum of the weights. Smaller WSD value indicates clustered distribution of influenza, on the other hand, larger one indicates dispersed distribution of influenza (see explanation in Figure 4). We excluded the week which only three or less prefectures reported the influenza cases, because the weighted mean center and distribution which was calculated based on the reported cases from three or less prefectures may not be meaningful. The compactness value can be represented on a map by drawing a circle with the radius equal to the value. Maps were for each season between the lowest WSD week and the peak week (named “early” and “late” respectively). The center of the circle is determined by the position of the each weighted mean center. All spatial analyses were performed by using ArcGIS 10 (ESRI, Redlands, CA) which has a function to calculate WSD as a standard tool in Arctoolbox and it provides how to work the function as references:

http://help.arcgis.com/en/arcgisdesktop/10.0/help/index.html#//005p0000001v000000 and

http://help.arcgis.com/en/arcgisdesktop/10.0/help/index.html#//005p0000001m000000.

### Statistical analyses

An adjusted 5-week un-weighted moving average of WSD values was used to evaluate the significance of WSD trends. WSD trends were then analyzed with influenza case trends to examine a possible relationship between those two trends. This was done by setting two time points; the first time point was the lowest WSD value week, which means the most compact distribution of an influenza outbreak (i.e. locally clustered outbreak); the second time point was the peak week of reported influenza cases by IDWR. The calculated duration between these two time points were evaluated to determine the significant association with the circulating influenza virus types/subtypes in the early phase of epidemic. Early phase was defined as period from the beginning of continuous report of influenza virus as laboratory confirmed case to the peak week of influenza epidemic. Data of proportions of circulating influenza virus types/subtypes were calculated from reported number of laboratory confirmed cases divided by type and subtype based on information by IASR. Spearman’s correlation coefficient was employed to analyze the relationship between those two parameters. All analyses were done retrospectively. All calculations were done with Microsoft Excel 2007 (Microsoft Corp., Redmond, WA).

## Abbreviations

WSD: Weighted Standard Distance; IDSC: Infectious Disease Surveillance Center; IDWR: Infectious Diseases Weekly Report; IASR: Infectious Agents Surveillance Report.

## Competing interests

The authors declare that they have no competing interests.

## Authors’ contributions

YS performed analyses and wrote the manuscript with editing by RS, and HS. KT contributed to establish national surveillance system. TS and SI contributed to the discussion and improved the manuscript. SAW supervised the analyses. All authors read and approved the final manuscript.

## Supplementary Material

Additional file 1 Animated clip of the progression of WSD values overtime. Click here for file
